# Blackcurrant (*Ribes nigrum* L.) Seeds—A Valuable Byproduct for Further Processing

**DOI:** 10.3390/molecules27248679

**Published:** 2022-12-08

**Authors:** Magdalena Wójciak, Barbara Mazurek, Katarzyna Tyśkiewicz, Małgorzata Kondracka, Grażyna Wójcicka, Tomasz Blicharski, Ireneusz Sowa

**Affiliations:** 1 Department of Analytical Chemistry, Medical University of Lublin, Chodźki 4a, 20-093 Lublin, Poland; 2 Analytical Department, Łukasiewicz Research Network—New Chemical Syntheses Institute, Aleja Tysiąclecia Państwa Polskiego 13a, 24-110 Pulawy, Poland; 3 Department of Pathophysiology, Medical University of Lublin, Jaczewskiego 8b, 20-090 Lublin, Poland; 4 Department of Rehabilitation and Orthopaedics, Medical University of Lublin, 20-059 Lublin, Poland

**Keywords:** blackcurrant residues, blackcurrant seeds, fruit oil, supercritical fluid extraction

## Abstract

The rational exploitation of byproducts is important from the point of view of their potential applicability in various fields. In this study, the possibility of further processing of blackcurrant seeds (BCs), which are a byproduct of fruit processing, was investigated. BCs were used as a material for the extraction of oil on a semi-industrial scale, and the residues were assessed in terms of their potential application in skin care products. Supercritical fluid extraction (SFE) using CO_2_ at pressures of 230 and 330 bar and extraction temperature of 40 °C was exploited for isolation of oil, and the products were characterised taking into account lipophilic constituents. After 120 min, the oil yields were 19.67% and 20.94% using CO_2_ at 230 and 330 bar, respectively, which showed that SFE was an effective method on a semi-industrial scale, taking into account the extraction yield. The oils had similar fatty acid compositions with a high percentage of linoleic acid (ca. 43%); however, tocopherols and carotenoids were most abundant in the oil obtained at 230 bar. It was also found that the composition of the SFE oils was comparable with that of cold-pressed oil, which shows that supercritical fluid extraction provides a high-quality product; therefore, it can be an alternative to cold pressing. Furthermore, the chemical compositions of the extracts from the oil isolation residues were established using UPLC-MS, and the impact of the extracts on human skin fibroblasts was assessed using the MTT and NR assays. The quantitative analysis revealed that the residues contained high amounts of polyphenolic acids, including gallic, protocatechuic, and hydroxybenzoic acid derivatives, as well as flavonoids, especially quercetin and kaempferol glucoside. Moreover, it was found that the extracts were nontoxic and exerted a stimulatory effect on cell metabolism. Therefore, they can be a valuable additive to natural plant-based cosmetics. Our results showed that blackcurrant seeds, regarded as a byproduct, can be a valuable material for further use.

## 1. Introduction

Blackcurrant (*Ribes nigrum* L.) berries are valuable seasonal fruits widely used for the manufacture of food products, such as beverages, juices, and jams, and for direct consumption. They are commonly known to have many valuable components, including phenolic acids, flavonoids, and anthocyanins. They are also rich in vitamins, especially ascorbic acid [1]. Seeds that remain after berry processing are regarded as unwanted waste; however, they can be a valuable product for further processing, which is in line with the current trend of “zero waste”. Such a byproduct can be a source of high-value biomolecules useful as an additive to functional foods, cosmetics, and pharmaceutical preparations [2].

Blackcurrant seeds are a source of oil (BCo) that is characterised by a desirable fatty acid composition with high content of polyunsaturated fatty acids (PUFAs) and a favourable n-6/n-3 ratio [3,4]. Moreover, it contains other important components, such as tocopherols, carotenoids, and phytosterols [5]; therefore, it can be an attractive dietary supplement with significant health benefits [6,7,8]. Some reports have demonstrated that it has a positive effect on the metabolism of lipids [6] and shows immunomodulating activity [7]. Capsules with blackcurrant oil decrease the concentration of lipids in serum, and dietary supplementation therewith prevents hypertension and atopic dermatitis [8,9,10]. In mentioned reports, BCo was obtained using solvent extraction or pressing.

Solvent extraction is very effective; however, nowadays this technique is mainly used on a laboratory scale because organic solvents contribute to environmental pollutants, and their residues found in the oil are harmful to humans. Mechanical extraction with the use of screws or hydraulic presses is the most common method applied for oil extraction on an industrial scale [11]. Pressing can yield a valuable product; however, it has some limitations. One of them is low profitability in the case of plant material containing less than 15% of oil [12]. The other drawbacks are the relatively low efficiency of extraction and time consumption of the process, which can take up to several hours. Moreover, the process takes place in atmospheric conditions; thus, the obtained product is susceptible to oxidation [13]. Taking into consideration the abovementioned aspects, supercritical carbon dioxide extraction (scCO_2_) seems to be a promising alternative for oil production. It is regarded as environmentally friendly as it does not generate harsh pollution and yields high-quality oil free from organic residues. Moreover, extraction with supercritical fluids is often carried out at relatively low temperatures, without access to oxygen, which prevents thermal and oxidative changes in bioactive compounds [14]. The scCO_2_ technique can also be used to design oils with a desirable composition, enhancing the nutritional properties of the product [15]. scCO_2_ extraction has been considered as a tool for analytical purposes only [16], but in recent years it has gained greater significance for industrial purposes [17].

The goal of the study was to investigate the potential of blackcurrant seeds, which are commonly regarded as a waste product, as a material for further processing to obtain high-quality oil using SFE on a large scale. The quality of the oil was assessed in subsequent extraction stages to assess the changes in the chemical composition during the process, which yielded a product with high contents of favourable lipophilic components in a relatively short time. The composition of the SFE oil was compared with oil obtained by cold pressing, as the method used may significantly affect the chemical composition and as it is often believed that the process carried out on a large scale provides a poor-quality product.

Furthermore, we aimed to establish the phytochemical profile of solid residues after oil extraction and the effectiveness of isolation of polyphenols from the residues using a safe and skin-friendly extrahent in order to obtain a polyphenolic-rich fraction possible to use in skin care products. The impact of the extract on the viability of human skin fibroblasts was also assessed.

## 2. Results

### 2.1. Extraction and Chemical Composition of the Oil

**Figure 1:**
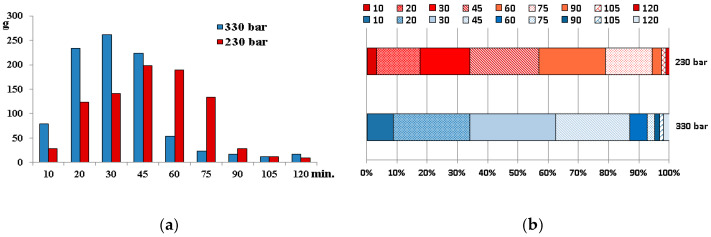
Amount of oil extracted in the particular stages (**a**) and percent contribution of the fractions in the final weight of the oil (**b**).

The temperature, time, pressure, and CO_2_ flow rate are the main factors influencing the effectiveness of supercritical fluid extraction. In our study, the temperature of the extraction was set at 40 °C to avoid degradation of thermolabile components, and the CO_2_ flow rate was established at the maximal level to accelerate the extraction. The process was conducted using two different pressures: 230 and 330 bars, oil fractions were collected every 10–15 min, and the effectiveness of extraction was assessed. Total extraction yields after 120 min of extraction were 19.67% and 20.94% using CO_2_ at pressures of 230 and 330 bar, respectively. However, the extraction efficacy and, therefore, the amount of oil extracted in the particular stages varied significantly (Figure 1).

The fractions had different colours, from light yellow to deep green (Supplementary Materials: Figure S1), depending on the stage of extraction and pressure used. The total content of pigments was assessed spectrophotometrically, and the results of HPLC quantification of lutein in the analysed fractions are presented in Table 1. A representative chromatogram is shown in Figure S2.

As can be seen, the highest total content of pigments was noted during the first 10 min of the process. Then, the concentration consequently decreased and increased again after 45–60 min of extraction but did not reach the initial level. Fraction F1 obtained at 230 bar had the highest contents of carotenoids and contained 17.5 mg of lutein and 1.8 mg of ß-carotene in 100 g of oil. In the other fractions, ß-carotene was not detected. High differentiation of the pigment profile between the fractions was observed, and the percent distribution of chlorophylls and carotenoids in the total pigment content varied significantly (Figure S3), which affected the colour of the product. The percent contribution of carotenoids in the oil obtained at 230 bar was higher and ranged from 4.6 to 12.5% in fractions F1 and F7-9 and from 26 to 42.6% in the other fractions, which determined the greenish and yellow colour of oil, respectively. The carotenoid content in the fractions obtained at 330 bar did not exceed 24.3%.

Similar to pigments, the concentration of tocopherols in the fractions varied significantly depending on the conditions of extraction and the stage of the process. The results of the HPLC analysis are shown in Table 2. Three main peaks corresponding to δ, γ, and α tocopherols were visible in the FL chromatograms of the oil sample (Figure S4), and γ tocopherol was dominant. The highest amount of tocopherols was noted in fractions F1–F2; next, the content decreased but increased slightly in the final extraction steps. However, the percent distribution of tocopherols was similar in all the fractions (Figure S5). γ tocopherol was the most abundant, and its percent contribution was ca. 65–75% of all tocopherols, whereas α tocopherol was present at the lowest concentration of 2–6.2%. Only in fraction F1 (230 bar), the level of α tocopherol was higher and reached ca. 13%.

In contrast, the concentration of fatty acids and the profile of the main fatty acids were similar in all oil fractions, except for fraction F1 obtained using CO_2_ at 230 bar, which had a significantly lower amount of linoleic, oleic, and γ-linolenic acid (Table 3). In the other fractions, linoleic acid concentration ranged from 36.1% to 46.3%, followed by γ-linolenic acid (from 12.3 to 17.4%), α-linolenic acid (from 10.4 to 14.1%), and oleic acid (from 7.2 to 14.9%). The content of saturated acids was below 10%, and the total content of FA was in the range of 76.6–99.7%. Noticeably higher contents of γ-linolenic acid and total FAs were found in fractions F3–F5 obtained at 230 bar.

### 2.2. Characteristics of the Oil Obtained Using SFE and Cold Pressing

The final products from SFE extraction and cold-pressed oil were characterised in terms of the total content of pigments as well as the content of the main components, such as fatty acids, tocopherols, and carotenoids. The results are summarised in Table 4.

As can be seen, the highest differentiation was noted in the case of pigments. The product obtained at 230 bar contained more carotenoids than the oil obtained at the higher pressure, and carotenoids accounted for ca. 16% of the total amount of pigments. Moreover, the total amount of chlorophyll was lower, which positively influenced the colour of the product. On the other hand, in cold-pressed oil, no chlorophyll was found, and total amount of tocopherols was slightly higher (approx. 7%) than in oil obtained at 230 bar. In turn, irrespective of the extraction type, the composition of fatty acids did not differ significantly; linoleic acid was the most abundant and comprised ca. 43%, followed by γ-linolenic and α-linolenic acid (ca. 16 and 13%, respectively), and the content of saturated fatty acids was ca. 8.5%. It should also be mentioned that effectiveness of cold pressing was significantly lower than SFE extraction. It yielded 12.9% of the oil.

### 2.3. Extracts from Solid Residues after Oil Extraction

#### 2.3.1. UPLC Analysis

**Figure 2:**
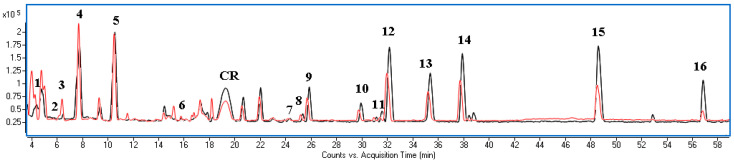
Overlapped BPC chromatograms of extracts obtained using: methanol/water 8:2 *v/v* (grey line) and water/1,3-propanediol 6:4 *v/v* (red line). Numbering of components as in Table 5. CR—cyanidin rutoside.

Phenolic compounds in the extracts were identified using UPLC-MS based on comparison mass data and UV-Vis spectra with the literature [18,19,20] and standards when available (Table S1). Phenolic acids were predominant compounds in analysed extracts, and gallic acid, protocatechuic acid, and p-hydroxybenzoic acid and its hexoside were the most abundant. Identified flavonoids belonged to myricetin, quercetin, and kaempferol derivatives. One compound belonging to anthocyjans with characteristic maximum absorbance at the visible region was also found in the extracts. The ionisation in the positive mode showed the parent ion at *m*/*z* + H 595.16594 (generated formula: C_27_H_31_O_15_+, Δppm 0.33) and an additional pseudomolecular ion *m*/*z* + H 287, and based on the literature [18], it has been identified as cyanidin 3-O-rutinoside. Methanol with water (8:2, *v*/*v*) was used to establish the total amount of the polyphenols in residues as it is the most recommended extrahent for polyphenolic compounds. In turn, the application of water with 1,3-propanediol for extraction allowed assessment of the suitability of BC residues as a material useful for cosmetic purposes. It was found that the BC residues were a particularly rich source of gallic and protocatechuic acids and glucosides of quercetin and kaempferol (Table 5). The qualitative profile of polyphenols in all extracts was similar (Figure 2); however, the quantity of the components varied. The water/1,3-propanediol mixture was more effective for the extraction of highly polar components such as gallic and protocatechuic acid as well as dihydroxy- and hydroxybenzoic acid hexoside. On the other hand, the methanol/water mixture more effectively isolated a less polar aglycone form.

It should also be mentioned that some phenolic compounds in low amounts were also found in the initial fraction of SFE oils (Figure S6).

#### 2.3.2. Cell Viability Assay

**Figure 3:**
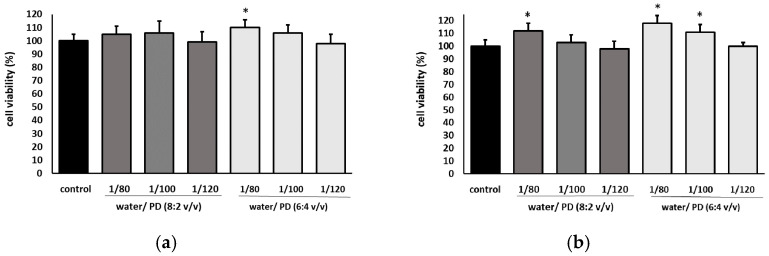
Effect of the different dilutions of water/1,3-propanediol extracts from blackcurrant seed residues on cell viability determined by the NR (**a**) and MTT (**b**) assays expressed as a % of control (0.5% of 1,3-propanediol in medium). The data are means ± SD (n = 3). One-way ANOVA followed by Dunnett’s post hoc test was conducted; the differences were considered significant at *p* ≤ 0.05. * indicates statistically significant difference. PD—1,3-propanediol.

The effect of the water/1,3-propanediol extract from blackcurrant seed residues on the fibroblast cells was assessed using two complementary tests: neutral red and MTT assays (Figure 3). The first one allows assessment of the stability of cell membranes, and the second shows the impact on cellular metabolism. As can be seen, the tested extracts did not decrease cell viability; therefore, they were nontoxic. Moreover, at the dilution of 1:80 and 1:100 ***v***/*v,* they even showed stimulatory effects on cell metabolism. It was also noted that the extracts did not affect the cell morphology (Figure S7).

## 3. Discussion

Nowadays, the European community pays great attention to reusing wastes, and many of them are regarded as valued bioresources. Fruit processing generates significant amounts of solid residues, including seeds. They are considered undesired byproducts; however, they can be valuable materials for further processing [2,21].

Our study showed that blackcurrant seeds may be a source of oil with beneficial prohealth features and that residues after oil extraction are rich in polyphenolic compounds. Two environmentally friendly techniques were applied for oil extraction including SFE and cold pressing, and it was shown that the method used had great importance as it affected the chemical composition of the product. It should be noted that SFE at a lower pressure yielded a product with better quality than oil isolated at 330 bar. Pressure is one of the key parameters in extraction using supercritical CO_2_. An increase in pressure is associated with an increase in fluid density; therefore, it generally should enhance the solubility of the components. However, above a certain value, the diffusivity of the SCF solvent may be lowered and, as a result, contact with the surface of the material is lower, which may decrease the transfer of compounds from the matrix. Moreover, at increased pressure, the material may be compressed, which reduces the surface of contact and leads to a deterioration of the efficiency of extraction of particular components, which may explain the results of our study. Since a slight decrease in the content of tocopherols, lutein, and unsaturated fatty acids was observed at 330 bar, we decided not to continue the experiment with higher pressure.

As can be seen, the SFE oil contained more pigments and lutein compared to cold-pressed oil, and in turn, the second one had a higher content of α tocopherol. Plant pigments are natural antioxidants increasing oil stability, and carotenoids are important components of human diet, e.g., ß-carotene is a precursor of vitamin A and lutein plays an important role as a protector of the macula [22]. On the other hand, α tocopherol is the most active form of tocopherols, and it is a highly desirable component of the oils [23].

Regardless of the technique of extraction, BC oils have beneficial fatty acid profiles with a high content of unsaturated acids, including γ-linolenic and α-linolenic acids, which are not produced in the human organism and need to be supplied with diet. They are essential for growth and development and have a beneficial impact on human health. For example, it has been shown that γ-linolenic acid improves the lipid profile in blood, alleviates osteoporosis symptoms, and is helpful in skin disorders [24].

The effectiveness of oil extraction from blackcurrant seeds depends on the method used. According to the literature (Table 6), the yield may vary from a low percentage to even thirty percent, and solvent extraction is the most effective. The yields obtained in our study using the quarter-technical installation were significantly higher than values reported in the literature for SFE of blackcurrant oil. Gustinelli et al. [16], who conducted extraction on a laboratory scale, obtained only 2 and 6% of oil, whereas Rój et al. [25] obtained 13–17% of the product after 240 min of extraction. However, it should be mentioned that the CO_2_ flow rates in both studies were three- and two-fold lower, respectively, than in our investigations.

Comparing to literature data in terms of the chemical composition of BC oil, it can be seen that the contribution of particular tocopherol forms varied; however, no relationship between the tocopherol profile and the method of extraction was observed. A similar pattern of tocopherols to that obtained in our study was found in cold-pressed oil [4]. In contrast, the amount of α and γ tocopherols was almost equal in the SFE oil obtained by Gustinelli et al. [16]. On the other hand, similar to our findings, in oils obtained by Mildner-Szkudlarz et al. [26] and Goffman et al. [27], γ tocopherol was the most abundant component (ca. 63%), but the amount of α-tocopherol was a few-fold higher than that of δ tocopherol. The differences may be a result of different profiles of the plant material. The reported profile of fatty acids was similar in a majority of papers. According to the literature, linoleic acid was the most abundant compound in BC oil (40 to 48% of total fatty acids), followed by γ-linolenic acid (11–16%), oleic acid (11–14%), palmitic acid (4.5–9.6%), and a low amount of stearic acid (1.4–2%). Our results are within these ranges.

**Table 6:** Comparison of blackcurrant oil obtained using different extraction methods.

Method	Yield (%)	Tocopherol (mg/100 g)			Fatty Acid (% *w*/*w*)						Ref.
		δ	γ	α	Palmitic	Stearic	Oleic	Linoleic	γ-Linolenic	α-Linolenic	
pressing	18.2	12.4	78.1	33.1	5.76	1.37	13.43	48.15	16.19	11.93	[26]
pressing	16.2	84.3	117.9	18.4	9.63	1.39	12.09	38.64	18.54	13.57	[4]
SE	15.9	-	-	-	5.2	1.8	10.3	48.2	11.3	17.5	[28]
SE	25.5–29.2	-	-	-	4.1–5.5	1.2–2.3	11.1–12.2	40.6–45.3	16.2–18.8	12.9–16.2	[29]
SE	ca. 10	12.5	69.7	30.7	7.7	2.4	12.9	44.1	12.8	14.5	[16]
Soxhlet	26.15	4.09	23.0	28.8	4.49	1.93	13.79	41.41	14.89	12.91	[3]
Soxhlet	20.3	8.58	103.3	59.7		-	-	-	13.8	-	[27]
Soxhlet	14.5	-	-	-	5.9	1.4	9.3	44.6	12.6	12.2	[30]
SFE 50 °C SFE 80 °C	ca. 2 ca. 6	16.0 13.7	102.7 87.4	122.1 93.2	7.9 7.7	2.1 2.1	12.3 13.0	42.5 42.1	13.4 12.7	16.4 17.5	[16]

SE—solvent extraction, supercritical fluid extraction on laboratory scale.

Paying attention to the economics of the process of extraction of plant materials using scCO_2_, it should be noted that the cost of the SFE equipment is relatively high. In addition, experience of the operator and technical knowledge are required, as several factors, e.g., temperature, pressure, and CO_2_ flow rate, must be taken into account to ensure a high-quality product combined with profitable efficiency. However, the short time and high extraction efficacy as well as the lack of air access during oil isolation, which reduce the risk of lipid oxidation, compensate for these disadvantages and make SFE a promising alternative to cold pressing. It should also be emphasised that although the cost of the apparatus is high, the cost of oil production depending on the type and the amount of plant material used may be low and even comparable to that of cold pressing. CO_2_ is inexpensive, and the energy consumption is low because the SFE process is generally faster. Moreover, the resulting oil is free from residues, and no refining is needed [11,31].

Our study also showed that solid residues retained after oil extraction could be valuable materials for further processing as they were rich in polyphenolic compounds known for their multidirectional activity and high antioxidant potential. Gallic acid and protocatechuic acid followed by quercetin glucoside were the most abundant. They have anti-inflammatory and free-radical-scavenging activities [32,33,34,35], and these features are highly desirable in skin care products because oxidative stress promotes unfavourable processes in the skin, including degradation and disorganisation of collagen fibres that promote skin ageing [36].

Moreover, water with 1,3-propanediol was an effective extrahent to isolate polyphenols from seed residues. Although the extraction efficiency was slightly lower than for the methanol/water mixture, it should be noted that in contrast to methanol, 1,3-propanediol is a permitted solvent for direct usage on skin. It is well tolerated, enhances transdermal absorption of many ingredients and the retention of moisture in the skin, and acts as an emollient and preservative in skin care products; therefore, propanediol/water extract can be a valuable additive to natural cosmetics. It is worth noting that such extracts were nontoxic for human fibroblast cells and even stimulated cell metabolism as shown in our study.

To sum up, our study has demonstrated that the BC seeds regarded as an unwanted byproduct can be a valuable material for further processing

## 4. Materials and Methods

### 4.1. Reagents and Standards

Analytical standards and reagents, including fatty acid methyl esters (FAMEs), polyphenolic compounds, tocopherols, lutein, ß-carotene, trimethylsulfonium hydroxide (TMSH) solution (∼0.25 M in methanol), 2,2-diphenyl-1-picrylhydrazyl (DPPH), Folin–Ciocalteu reagent, and trolox were purchased from Fluka (Sigma-Aldrich Co., St. Louis, MO, USA).

Methanol, acetonitrile, isopropanol, trifluoroacetic acid, and LC-MS-grade formic acid were purchased from Merck KGaA (Darmstadt, Germany). Acetonitrile Optima^®^ LC-MS grade was purchased from Fischer Chemical (Waltham, MA, USA). Tert-butyl methyl ether 99.8% was purchased from Avantor Performance Material, Poland. Water was deionised and purified using Ultrapure Millipore Direct-Q^®^ 3UV-R (Merck, KGaA, Germany).

### 4.2. Plant Material

The blackcurrant seed was obtained from a local Polish juice manufacturer (Lublin province) as a mix of seeds from different cultivars of blackcurrant growing in eastern Poland in 2019–2020. The cultivars commonly used for juice production in Poland are as follows: Polonus, Tiben, Polares, Gofert, and Tisel. The fresh residues were treated according to Milala et al. [37]. Prior to extraction, seeds were crushed using roller mill crusher (Sipma H-752, Poland) equipped with crushing rollers with a diameter of 240 mm. Particle sizes after crushing were approx. 0.4–0.6 mm.

### 4.3. Extraction of Oil

The extraction of seeds was carried out in the Łukasiewicz Research Network—New Chemical Syntheses Institute in Puławy (Poland) using an in-house-built quarter-technical installation. The scheme is presented in Figure S8. After crushing, the raw material was placed in the extractor. Liquid CO_2_ from the reservoir, compressed to the assumed extraction pressure using the pump, was heated in the heat exchanger to reach the assumed temperature. Obtained in this way, the supercritical CO_2_ was directed to the extractor filled with blackcurrant seeds.

The process in continuous mode was conducted at 40 °C, two different pressures of 230 and 330 bar, and a CO_2_ flow rate of 145 kg/h and with a total extraction time of 120 min. The weight of seeds taken for loading of the extractor was ca. 4390 ± 0.02 g. The samples were taken every 10–15 min. Oil samples were stored in a fridge until analysis. Cold-pressed oil was obtained using Yoda plus YD-ZY-02A (YODA, Bielsko-Biala, Poland).

### 4.4. Total Chlorophyll and Carotenoid Assay

Total chlorophyll and carotenoid contents were assessed based on the method proposed by Wellburn [38]. A total of 50 mg of oil was mixed with 1 mL of isopropanol, vortexed for 5 min at 3000 rpm, and centrifuged at 6000 rpm. Spectrophotometric measurements were carried out at 470, 646, and 663 nm for carotene, chlorophyll b, and chlorophyll a, respectively.

### 4.5. GC Determination of Fatty Acids (FAs)

The GC methodology was based on the literature [39] with a slight modification. Samples were prepared as follows: 10 (±2) mg of the sample was dissolved in 500 μL of tert-butyl methyl ether, mixed with 250 μL of the trimethylsulfonium hydroxide solution (0.25 M in methanol), and shaken for several minutes to obtain methyl derivatives (FAMEs). The analysis was performed on a gas chromatograph with a single quadrupole mass spectrometer (GC/MSD 6890N/5975) and a split/splitless injector controlled by MSD ChemStation, version E.02.02.1431 (Agilent Technologies, Inc., Santa Clara, CA, USA). Capillary column type HP-88 (60 m, 0.25 mm i.d., and 0.20 μm) with an 88% cyanopropylaryl polysiloxane phase (Agilent) was used for separation, and 1 μL of the sample was injected. The split ratio was 150:1, the split flow rate was 180 mL/min, and the helium (carrier gas) flow rate was 1.2 mL/min. The oven temperature was started at 110 °C and maintained for 2 min. Next, it was increased to 190 °C at a rate of 8 °C/min and maintained at 190 °C for 13 min. The MSD parameters were as follows: scan mass 50–500 amu, EM voltage 1550, MS source temperature 230 °C, and MS quad temperature 150 °C. FAMEs were identified by comparing their mass spectra, fragmentation patterns, and retention time peaks with appropriate standards and with the NIST library. The FA contents are expressed as g FA/100 g of sample (% *w*/*w*).

### 4.6. Chromatographic Analysis

#### 4.6.1. Tocopherols and Carotenoids

The oil samples (0.04–0.05 g) were diluted in 1 mL of isopropanol, vortexed for 5 min at 3000 rpm, and centrifuged at 6000 rpm. The quantitative chromatographic analyses were performed using VWR Hitachi Chromaster 600 chromatograph equipped with a fluorescent (FL) and spectrophotometric detector (PDA), a thermostat, and EZChrom Elite software (Merck, Darmstadt, Germany) on an RP18 reversed-phase column. The chromatographic conditions for tocopherols were based on the literature [40,41] with a slight modification. The mobile phase was a mixture of methanol and acetonitrile (95:5, *v*/*v*), the flow rate was 1.2 mL/min, and the temperature of the thermostat was set at 30 °C. Carotenoids, including lutein, were eluted using methanol and isopropanol (95:5, *v*/*v*) as an eluent. The flow rate was 1 mL/min, and the thermostat temperature was 30 °C.

#### 4.6.2. Phenolic Constituents

A total of 1 g of solid residues after the extraction of oil at 230 bar was extracted using 10 mL of the methanol/water mixture (8;2, *v*/*v*) in a water bath at 80 °C for 40 min. The process was repeated three times with fresh portions of solvent, and extracts were combined and concentrated to a final volume of 10 mL (total extraction).

A total of 1 g of solid residues after the extraction of oil at 230 bar was extracted using 10 mL of the water/1,3-propanediol mixture (8:2, and 6:4, *v*/*v*) in a water bath at 80 °C for 40 min.

For the analysis of polyphenols in the oil, the samples were prepared according to the procedure proposed by Becerra-Herrera et al. [42]. A total of 0.050 g of oil was extracted using 1 mL of a methanol/water mixture (8:2, *v*/*v*), vortexed for 5 min at 3000 rpm, and centrifuged at 6000 rpm. The upper fraction was collected and analysed.

Polyphenolic components were analysed using an ultra-high performance liquid chromatograph (UHPLC) Infnity Series II with a DAD detector and Agilent 6224 ESI/TOF mass detector (Agilent Technologies, Santa Clara, CA, USA). Chromatographic conditions were as follows: an RP18 reversed-phase column Titan (Supelco, Sigma-Aldrich, Burlington, MA, USA) (10 cm × 2.1 mm i.d., 1.9 µm particle size), a thermostat temperature of 30 °C, and a flow rate of 0.2 mL/min. The mobile phase consisted of water with 0.05% of formic acid (solvent A) and acetonitrile with 0.05% of formic acid (solvent B). The gradient elution program was as follows: 0–8 min from 98% A to 93% A (from 2% to 7% B), 8–15 min from 93% A to 88% A (from 7% to 12% B), 15–29 min from 88% A to 85% A (from 12% to 15% B), 29–40 min from 85% A to 80% A (from 15% B to 20% B), and 40–60 min from 80% A to 65% A (from 20% B to 35% B) [43]. Chromatograms were recorded from 200 to 600 nm. LC–MS analysis: the ion source operating parameters were as follows: drying gas temperature 325 °C, drying gas flow 8 L min^−1^, nebuliser pressure 30 psi, capillary voltage 3500 V, and skimmer 65 V. Voltage on the fragmentator was 200 V. Ions were acquired in the range of 100 to 1300 *m*/*z*. MS identification was performed based on literature data.

### 4.7. Cell Culture

Human skin fibroblast cell lines (ATCC^®^ CRL-2522™) were from the American Type Culture Collection (Manassas, VA, USA). They were grown in Dulbecco’s Modification of Eagle’s Medium (DMEM, Biological Industries, Cromwell, CO, USA) with sodium pyruvate, L-glutamine, 10% fetal bovine serum (Gibco, Waltham, MA, USA), glucose (4.5 g/L), and 1% antibiotics (100 U/mL penicillin and 1000 µg/mL streptomycin, Gibco). Cultured cells were kept in a humidified atmosphere of 95% air and 5% carbon dioxide at 37 °C. When the cells obtained the required confluence, the medium was removed, and the cells were rinsed twice with sterile phosphate-buffered saline (PBS, Gibco). The confluent layer was trypsinised (0.25% Trypsin/EDTA, Gibco) and placed in fresh medium. Next, the cells were plated in 96-well flat-bottom plates and incubated for at least 24 h, and after that, they were treated with varying dilutions (1:80, 1:100, or 1:120 *v*/*v*) of the extracts for 24 h. The concentration of 1,3-propanediol was below 0.5% and did not affect the cells.

### 4.8. Cell Viability Assay

All experiments were performed in triplicates for each extract concentration and presented as a percentage of the control (100%). Cells treated with 0.5% of 1,3-propanediol in culture medium were taken as control.

#### 4.8.1. MTT Assay

The MTT assay is based on the reduction of a yellow tetrazolium salt (3-(4,5-dimethylthiazol-2-yl)-2,5-diphenyltetrazolium bromide—MTT) to purple formazan by NAD(P)H-dependent oxidoreductase enzymes in viable cells.

After 24 h of incubation of the cells with investigated extracts, an MTT solution (5 mg/mL) (Sigma) was added (25 μL/well), and further incubation was conducted for the next 3 h. The insoluble formazan crystals were solubilised overnight in a 10% sodium dodecyl sulfate (SDS) in 0.01 M HCl mixture. Absorbance was measured at the 570 nm wavelength using an E-max Microplate Reader (Molecular Devices Corporation, Menlo Park, CA, USA).

#### 4.8.2. Neutral Red Uptake Assay

The neutral red (NR) assay is based on the uptake of the dye via active transport, which is further cumulated in lysosomes of viable cells.

After 24 h of incubation of the cells with the analysed extracts, the neutral red dye at a concentration of 40 µg/mL was added to the wells. The plates were placed in an incubator for 2 h at 37 °C, the neutral red dye was removed, and the cells were washed with PBS. After this, PBS was removed, 150 µL of decolourising buffer was added, and the absorbance measurements were performed at wavelength λ = 540 nm.

### 4.9. Statistical Analysis

All analyses were performed in triplicates. The results were analysed using Statistic ver. 13.3 software. One-way ANOVA followed by Dunnett’s post hoc test was used.

## 5. Conclusions

The food industry generates high amounts of organic wastes. Searching for the possibility of the rational exploitation of the byproducts is important from the point of view of their potential applicability in various fields. These byproducts are easily available and cheap because they are problematic for food producers; however, they contain many components worth recovering. Our results show some perspectives for the application of this type of material as a source of valuable components for further use.

In this study, blackcurrant seeds, i.e., a byproduct of fruit processing, was used as a material for oil extraction and for obtaining a polyphenolic-rich fraction. Blackcurrant seed oil was obtained using the supercritical extraction technique on a semi-industrial scale. The process was characterised by high efficacy and yielded a high-quality product with a chemical composition similar to that of cold-pressed oil, which demonstrated that SCF can be a sustainable alternative to traditional cold pressing of oil.

Moreover, the extract obtained from the residues after oil isolation appeared to be an abundant source of polyphenolic acids and flavonoids, showed no cytotoxicity to human skin fibroblasts, and even stimulated cell proliferation. Therefore, it is a good candidate to be an additive to skin care products and is a promising basis for further investigation and development of natural plant-based products.

Future studies should focus on the elaboration of environmentally friendly methods for large-scale recovery of desirable components from byproducts, investigation of the potential of other materials regarded as byproducts, and prospects for new applications of byproducts in various fields.

## Figures and Tables

**Table 1:** Total content of pigments and content of lutein (mg/100 g) in the fractions of blackcurrant seed oils.

Component	F1	F2	F3	F4	F5	F6	F7	F8	F9
CO_2_ pressure: 230 bar									
chlorophyll A	392 ± 12.3 ^a^	25.6 ± 1.2 ^b^	3.2 ± 0.3 ^c^	1.8 ± 0.1 ^d^	1.3 ± 0.1 ^d^	5.3 ± 0.13 ^e^	40.7 ± 3.1 ^f^	57.6 ± 3.8 ^g^	60.9 ± 4.2 ^g^
chlorophyll B	5.6 ± 0.2 ^a^	0.5 ± 0.0 ^b^	0.1 ± 0.0 ^c^	0.3 ± 0.0 ^d^	0.4 ± 0.0 ^d^	0.9 ± 0.1 ^c^	4.0 ± 0.25 ^b^	13.7 ± 1.0 ^e^	18.4 ± 1.1 ^f^
carotenoids	19.0 ± 1.1 ^a^	13.9 ± 1.0 ^b^	2.4 ± 0.2 ^c^	1.2 ± 0.1 ^d^	0.8 ± 0.1 ^d^	2.2 ± 0.1 ^c^	6.4 ± 0.4 ^e^	9.4 ± 0.6 ^f^	10.2 ± 0.9 ^f^
lutein	17.5 ± 1.1 ^a^	12.8 ± 0.9 ^b^	2.2 ± 0.2 ^c^	1.1 ± 0.4 ^d^	ND	2.0 ± 0.1 ^c^	5.9 ± 0.3 ^e^	8.7 ± 0.7 ^f^	9.4 ± 0.8 ^f^
CO_2_ reassure: 330 bar									
chlorophyll A	248 ± 10.2 ^a^	2.7 ± 0.2 ^b^	2.0 ± 0.1 ^b^	12.0 ± 0.8 ^c^	41.7 ± 1.9 ^d^	55.6 ± 2.7 ^e^	52.6 ± 4.1 ^e^	46.8 ± 2.2 ^f^	42.1 ± 2.6 ^d,f^
chlorophyll B	41.9 ± 3.2 ^a^	1.2 ± 0.1 ^b^	0.5 ± 0.0 ^c^	1.6 ± 0.1 ^b^	5.4 ± 0.3 ^d^	11.7 ± 0.9 ^e^	10.3 ± 0.8 ^e^	7.5 ± 0.4 ^f^	6.0 ± 0.4 ^d^
carotenoids	3.7 ± 0.2 ^a^	1.1 ± 0.9 ^b^	0.8 ± 0.1 ^b^	2.7 ± 0.2 ^c^	5.5 ± 0.4 ^d^	7.8 ± 0.5 ^e^	7.3 ± 0.3 ^e^	6.2 ± 0.4 ^d^	5.7 ± 0.4 ^d^
lutein	3.2 ± 0.2 ^a^	<LOQ	ND	2.3 ± 0.1 ^b^	4.74 ± 0.3 ^c^	6.7 ± 0.5 ^d^	6.3 ± 0.3 ^d^	5.3 ± 0.4 ^c^	4.9 ± 0.2 ^c^

Different letters in the same line show significant differences (*p* < 0.05). ND—not detected.

**Table 2:** Content of tocopherols (mg/100 g) in the fractions of blackcurrant seed oils.

Component	F1	F2	F3	F4	F5	F6	F7	F8	F9
CO_2_ pressure: 230 bar									
δ tocopherol	38.7 ± 2.5 ^a^	36.0 ± 2.4 ^a^	33.4 ± 1.8 ^b^	30.2 ± 1.4 ^b^	10.1 ± 1.2 ^c^	6.2 ± 1.1^d^	11.3 ± 1.0 ^c^	12.0 ± 1.4 ^cd^	13.9 ± 1.2 ^d^
γ tocopherol	141 ± 4.7 ^a^	130 ± 4.4 ^b^	119 ± 5.2 ^c^	72.1 ± 2.4 ^d^	38.6 ± 1.2 ^e^	22.4 ± 2.0 ^f^	32.5 ± 1.3 ^g^	26.0 ± 1.1 ^f^	23.8 ± 1.8 ^f^
α tocopherol	66.8 ± 6.1 ^a^	55.9 ± 1.8 ^a^	28.7 ± 1.2 ^b^	9.9 ± 0.4 ^c^	3.3 ± 0.4 ^d^	3.8 ± 0.3 ^d^	1.6 ± 0.2 ^e^	0.8 ± 0.1 ^f^	0.7 ± 0.1 ^f^
total	246 ± 13 ^a^	219 ± 10 ^b^	181 ± 5.5 ^c^	112 ± 4.7 ^d^	52.1 ± 5.0 ^e^	32.4 ± 3.8 ^f^	45.4 ± 3.3 ^eg^	38.8 ± 4.1 ^fg^	38.4 ± 3.7 ^fg^
CO_2_ pressure: 330 bar									
δ tocopherol	27.7 ± 2.2 ^a^	28.6 ± 2.0 ^a^	19.0 ± 1.1 ^b^	13.8 ± 1.2 ^cd^	12.7 ± 0.9 ^c^	14.1 ± 0.9 ^cd^	13.5 ± 0.5 ^cd^	13.4 ± 0.5 ^cd^	14.7 ± 0.6 ^d^
γ tocopherol	103 ± 7.6 ^a^	113 ± 7.3 ^b^	73.0 ± 2.3 ^c^	58.2 ± 2.2 ^d^	25.0 ± 1.3 ^e^	67.0 ± 2.3 ^f^	64.8 ± 1.2 ^f^	62.0 ± 2.6 ^f^	64.9 ± 2.5 ^f^
α tocopherol	23.2 ± 1.4 ^a^	15.7 ± 0.6 ^b^	12.3 ± 0.8 ^c^	4.6 ± 0.2 ^d^	ND	ND	5.5 ± 0.4 ^e^	6.2 ± 0.5 ^ef^	6.7 ± 0.2 ^f^
Total	154 ± 11 ^a^	157 ± 5 ^a^	104 ± 5 ^b^	76.7 ± 3.8 ^c^	37.7 ± 2.3 ^d^	81.1 ± 3.8 ^ce^	83.9 ± 2.2 ^e^	81.8 ± 3.9 ^ce^	86.3 ± 2.5 ^ce^

Different letters in the same line show significant differences (*p* < 0.05). ND—not detected.

**Table 3:** Composition of fatty acids (% w/w) in the fractions of blackcurrant seed oils.

Fatty Acid	F1	F2	F3	F4	F5	F6	F7	F8	F9
CO_2_ pressure: 230 bar									
palmitic	6.5 ± 0.2 ^a^	7.7 ± 0.1 ^b^	7.9 ± 0.3 ^b^	7.4 ± 0.1 ^b^	6.7 ± 0.2 ^a^	5.3 ± 0.2 ^c^	5.4 ± 0.0 ^c^	5.9 ± 0.1 ^c^	5.6 ± 0.1 ^c^
stearic	0.98 ± 0.06 ^a^	1.36 ± 0.22 ^b^	1.4 ± 0.1 ^b^	1.4 ± 0.0 ^b^	1.6 ± 0.0 ^b^	2.0 ± 0.1 ^c^	1.9 ± 0.0 ^c^	1.7 ± 0.0 ^c^	1.5 ± 0.2 ^b^
oleic	7.18 ± 0.26 ^a^	11.1 ± 1.52 ^b^	12.7 ± 0.6 ^b^	12.8 ± 0.2 ^b^	13.9 ± 0.3 ^c^	14.9 ± 0.6 ^c^	13.5 ± 0.2 ^c^	13.2 ± 0.15 ^c^	11.7 ± 1.6 ^b^
linoleic	26.1 ± 0.39 ^a^	39.3 ± 2.81 ^b^	44.3 ± 1.2 ^c^	44.4 ± 0.4 ^c^	46.3 ± 1.3 ^c^	45.2 ± 1.9 ^c^	41.6 ± 0.4 ^b^	42.0 ± 0.59 ^b^	38.5 ± 3.0 ^b^
γ-linolenic	8.16 ± 0.11 ^a^	15.2 ± 1.21 ^b^	17.4 ± 0.6 ^c^	17.3 ± 0.2 ^c^	17.1 ± 0.6 ^c^	14.7 ± 0.8 ^b^	13.5 ± 0.6 ^e^	14.3 ± 0.1 ^b,e^	13.5 ± 1.6 ^e^
α-linolenic	10.3 ± 0.2 ^a^	12.2 ± 1.5 ^b,e^	13.6 ± 0.4 ^c^	13.6 ± 0.1 ^cd^	14.1 ± 0.5 ^d^	13.0 ± 0.6 ^b,c^	12.0 ± 0.4 ^b,e^	12.4 ± 0.1 ^b,e^	11.5 ± 1.53 ^e^
total	59.2 ± 0.4 ^a^	86.8 ± 2.9 ^b,e^	97.2 ± 1.3 ^c,d^	96.9 ± 0.6 ^c^	99.7 ± 1.3 ^d^	95.1 ± 1.9 ^c^	87.9 ± 0.4 ^b^	89.6 ± 0.6 ^b^	82.3 ± 3.1 ^e^
ratio n–6/n–3	3.33	4.45	4.54	4.53	4.50	4.59	4.57	4.55	4.52
CO_2_ pressure: 330 bar									
palmitic	6.9 ± 0.1 ^a^	7.4 ± 0.1 ^b^	6.1 ± 0.2 ^c,e^	6.1 ± 0.1 ^c,e^	5.1 ± 0.9 ^d^	5.4 ± 0.2 ^d^	6.4 ± 0.1 ^a,c^	6.1 ± 0.2 ^c,e^	5.7 ± 1.1 ^d,e^
stearic	1.4 ± 0.0 ^a^	1.5 ± 0.0 ^b,e^	1.4 ± 0.0 ^a^	1.7 ± 0.0 ^c^	1.4 ± 0.2 ^a,b^	1.4 ± 0.0 ^a,b^	1.6 ± 0.0 ^d^	1.5 ± 0.0 ^d,e^	1.6 ± 0.2 ^d^
oleic	10.9 ± 0.1 ^a^	12.3 ± 0.0 ^b^	10.9 ± 0.3 ^a^	13.8 ± 0.2 ^c^	11.3 ± 2.1 ^a^	11.3 ± 0.3 ^a^	12.7 ± 0.3 ^b^	11.9 ± 0.3 ^b^	11.0 ± 2.1 ^a^
linoleic	37.7 ± 0.1 ^a^	42.8 ± 0.1 ^b^	37.2 ± 0.7 ^a^	43.7 ± 0.8 ^b^	36.1 ± 2.2 ^a^	36.7 ± 0.7 ^a^	42.2 ± 0.5 ^b,c^	40.4 ± 0.9 ^c^	37.9 ± 2.9 ^a^
γ-linolenic	13.5 ± 0.2 ^a^	16.6 ± 0.4 ^b^	13.7 ± 0.3 ^a^	15.2 ± 0.4 ^c^	12.3 ± 2.1 ^d^	12.8 ± 0.4 ^ad^	15.2 ± 0.3 ^c^	14.6 ± 0.5 ^c,e^	13.7 ± 2.6 ^a,e^
α-linolenic	11.1 ± 0.1 ^a^	13.1 ± 0.1 ^b^	11.2 ± 0.3 ^a^	12.9 ± 0.3 ^b^	10.4 ± 1.9 ^a^	10.7 ± 0.4 ^a^	12.7 ± 0.2 ^b^	12.2 ± 0.4 ^b^	11.5 ± 2.2 ^a^
total	81.5 ± 0.12 ^a^	93.8 ± 0.1 ^b^	80.5 ± 0.7 ^a^	93.6 ± 0.8 ^b^	76.6 ± 2.2 ^c^	78.4 ± 0.8 ^a,c^	90.7 ± 0.6 ^b^	86.8 ± 0.9 ^c,d^	81.3 ± 3.1 ^a^
ratio n–6/n–3	4.62	4.52	4.55	4.57	4.64	4.62	4.53	4.49	4.48

Different letters in the same line show significant differences (*p* < 0.05).

**Table 4:** Characteristics of blackcurrant oil obtained using CO_2_ at pressures of 230 and 330 bar and cold pressing.

Component (mg/100 g)	SFE 230 Bar	SFE 330 Bar	Pressing
chlorophyll A	21.27 ± 0.51	31.70 ± 0.31	nd
chlorophyll B	1.08 ± 0.01	5.41 ± 0.02	nd
carotenoids	4.26 ± 0.11	2.29 ± 0.12	4.11 ± 0.08
δ tocopherol	22.1 ± 1.1	20.2 ± 1.2	24.3 ± 2.1
γ tocopherol	74.9 ± 2.2	79.0 ± 3.5	76.3 ± 3.5
α tocopherol	19.1 ± 1.2	10.9 ± 0.6	23.2 ± 2.0
total tocopherol	116.1±7.4	110.1±6.1	123.8 ± 8.4
lutein	3.92 ± 0.08	1.98 ± 0.06	4.12 ± 0.31
Fatty acid composition (% *w*/*w*)			
palmitic acid (C16:0)	6.90 ± 0.19	6.86 ± 0.21	7.06 ± 0.25
stearic acid (C18:0)	1.53 ± 0.01	1.59 ± 0.01	1.39 ± 0.10
oleic acid (C18:1 n-9)	12.96 ± 1.18	12.83 ± 1.06	12.97 ± 1.17
linoleic acid (C18:2 n-6)	43.39 ± 1.67	42.92 ± 1.27	44.02 ± 1.89
γ-linolenic acid (C18:3 n-6)	16.07 ± 1.10	15.68 ± 0.98	15.99 ± 1.08
α-linolenic acid (C18:3 n-3)	13.22 ± 0.87	12.86 ± 1.07	13.46 ± 1.12
ratio n-6/n-3	4.49 ± 0.23	4.56 ± 0.18	4.46 ± 0.26

**Table 5:** Amount of phenolic compounds (µg/g ± SD) isolated from blackcurrant seed residues using methanol/water 8:2 *v*/*v*, water/1,3-propanediol 8:2 *v*/*v,* and water/1,3-propanediol 6:4 *v*/*v*.

No	Component	MeOH/Water (8:2)	Water/PD (8:2)	Water/PD (6:4)
1	gallic acid	35.12 ± 1.91	59.09 ± 3.25	39.17 ± 1.28
2	hydroxybenzoic acid hexoside ^1^	10.27 ± 0.50	18.71 ± 0.91	16.05 ± 0.71
3	dihydroxybenzoic acid hexoside ^2^	47.45 ± 2.13	61.56 ± 2.93	65.09 ± 3.13
4	protocatechuic acid	81.20 ± 4.01	92.06 ± 4.65	89.81 ± 3.94
5	p-hydroxybenzoic acid	2.42 ± 0.12	2.51 ± 0.21	2.91 ± 0.15
6	caffeic acid	21.76 ± 1.21	20.18 ± 1.08	23.01 ± 1.41
7	salicylic acid	1.51 ± 0.08	1.22 ± 0.09	1.48 ± 0.06
8	myricetin rhamnosylhexoside ^3^	8.18 ± 0.43	7.79 ± 0.28	7.91 ± 0.38
9	myricetin-3-O-galactoside ^3^	64.29 ± 3.21	55.64 ± 3.98	58.20 ± 3.02
10	kaempferol hexoside	26.98 ± 1.21	21.12 ± 1.03	20.85 ± 1.81
11	quercetin-3-O-rutinoside	7.91 ± 0.32	7.02 ± 0.41	6.95 ± 0.28
12	quercetin-3-O-glucoside	121.83 ± 3.14	102.10 ± 4.01	107.81 ± 3.96
13	kaempferol-3-O-rutinoside	2.02 ± 0.20	1.74 ± 0.11	1.98 ± 0.14
14	kaempferol-3-O-glucoside	104.44 ± 4.21	68.41 ± 2.12	76.01 ± 3.01
15	quercetin	6.21 ± 0.21	2.39 ± 0.11	4.21 ± 0.19
16	kaempferol	1.96 ± 0.12	0.28 ± 0.03	1.18 ± 0.09

Quantification based on calibration curve for: ^1^ p-hydroxybenzoic acid, ^2^ dihydroxybenzoic acid, and ^3^ myricetin glucoside; PD—1,3-propanediol.

## Data Availability

Data are contained within the article or Supplementary Materials.

## Supplementary Materials

The following supporting information can be downloaded at: https://www.mdpi.com/article/10.3390/molecules27248679/s1, Figure S1: Blackcurrant seeds (a) and the example of samples of blackcurrant oil fractions obtained using CO_2_ at pressure of 230 bar (b) and pressure of 330 bar (c); Figure S2: Chromatogram of oil sample obtained using CO_2_ at pressure 230 bar (fraction F1) and representative UV-Vis spectra of identified compounds. 1- lutein, 2- ß-carotene; Figure S3: Percent distribution of pigments in oil fractions obtained using CO_2_ at pressure of 230 and 330 bar; Figure S4: FL-chromatogram of fraction F1 the oil obtained using CO_2_ at pressure 230 bar 1- δ tocopherol, 2- γ tocopherol, 3- α tocopherol; Figure S5: Percent distribution of tocopherols in oil fractions obtained using CO_2_ at pressure of 230 and 330 bar; Figure S6: BPC chromatogram of oil sample obtained using CO_2_ at pressure 230 bar (fraction F1). Numbering of components as in Table 3; Figure S7: Image of human skin fibroblasts (HSF) cells: (a) control, (b) after 24-hour incubation with water/1,3-propanediol mixture (8/2 *v*/*v*) at a dilution of 1/80, (c) after 24-hour incubation with water/1,3-propanediol mixture (6/4 *v*/*v*) at a dilution of 1/80. May Grunwald-Giemsa and acridine orange staining (200x magnification); Figure S8: Block diagram of in-house built quarter-technical installation used in the study; Table S1: UPLC-MS characteristic of compounds identified in methanol/water extract from blackcurrant seeds residues [18,19,20].

## Sample Availability

Samples of the oil are available from the authors.
