# Supplementary material for: Blackcurrant (Ribes nigrum L.) Seeds—A Valuable Byproduct for Further Processing
Source: Molecules. 2022 Dec 8;27(24):8679. doi: 10.3390/molecules27248679 (PMC9781738; doi:10.3390/molecules27248679)
Supplement: Supplementary file 1 [file molecules-27-08679-s001.zip › molecules-2035867-supplementary.pdf]

molecules

MDPI

Supplementary material

Blackcurrant (*Ribes nigrum* L.) Seeds—A Valuable Byproduct for Further Processing

Magdalena Wójciak [1,*], Barbara Mazurek[2], Katarzyna Tyśkiewicz[2], Małgorzata Kondracka[2], Grażyna Wójcicka[3], Tomasz Blicharski[4] and Ireneusz Sowa[1]

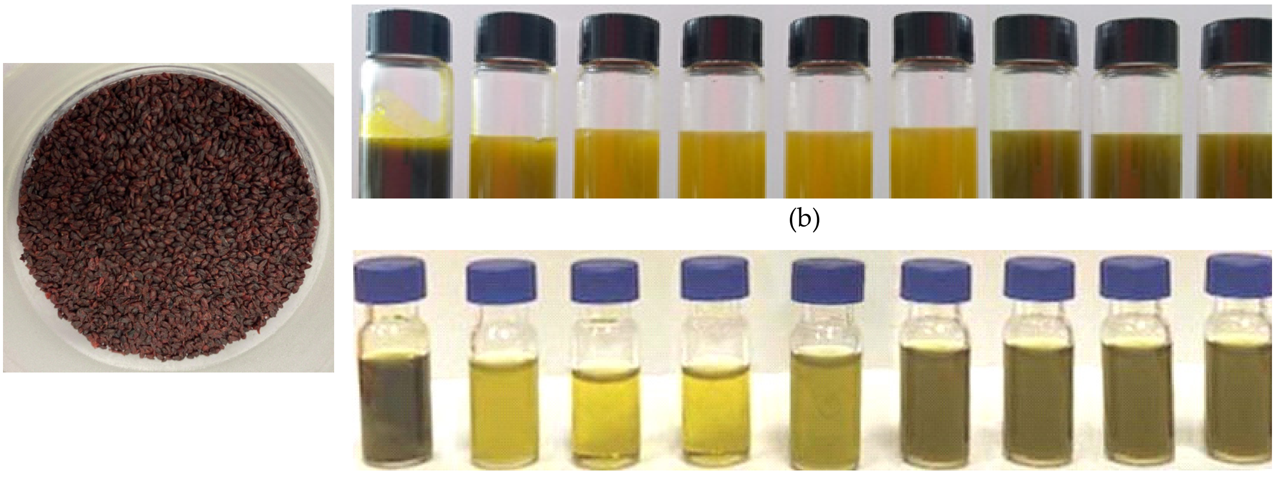

Figure S1. Blackcurrant seeds (a) and the example of samples of blackcurrant oil fractions obtained using CO_2 at pressure of 230 bar (b) and pressure of 330 bar (c).

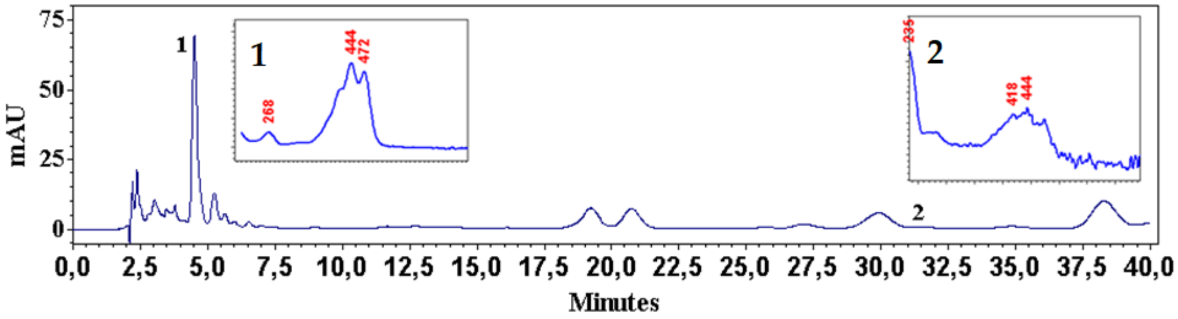

Figure S2. Chromatogram of oil sample obtained using CO_2 at pressure 230 bar (fraction F1) and representative UV-Vis spectra of identified compounds. 1- lutein, 2- ß-carotene

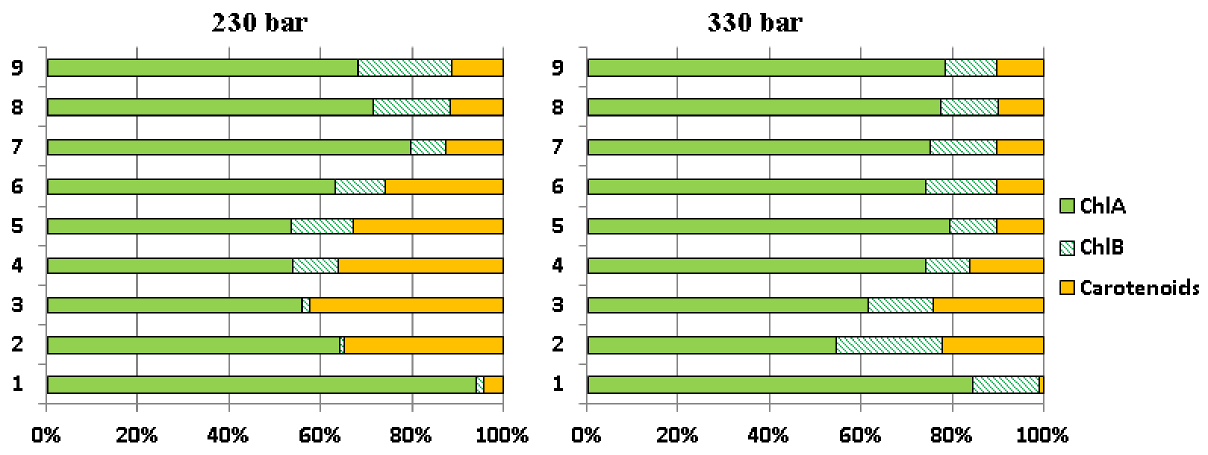

Figure S3. Percent distribution of pigments in oil fractions obtained using CO_2 at pressure of 230 and 330 bar.

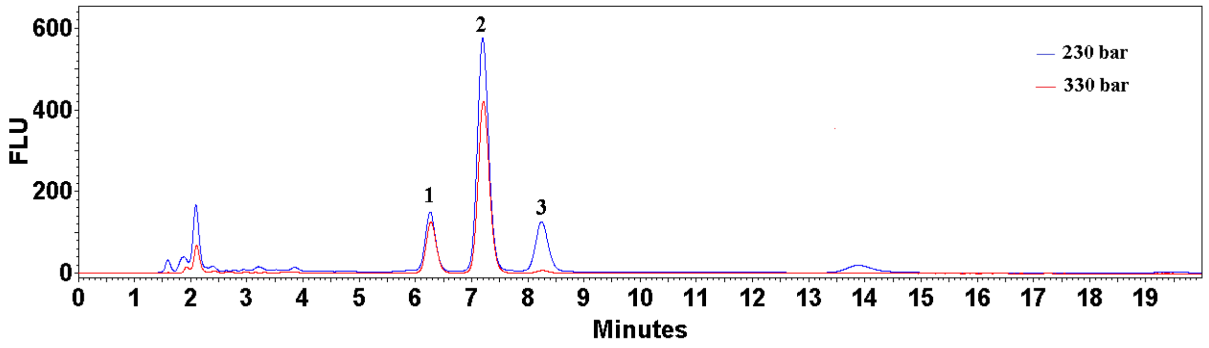

Figure S4. FL-chromatogram of fraction F1 the oil obtained using CO_2 at pressure 230 bar 1- δ tocopherol, 2- γ tocopherol, 3- α tocopherol

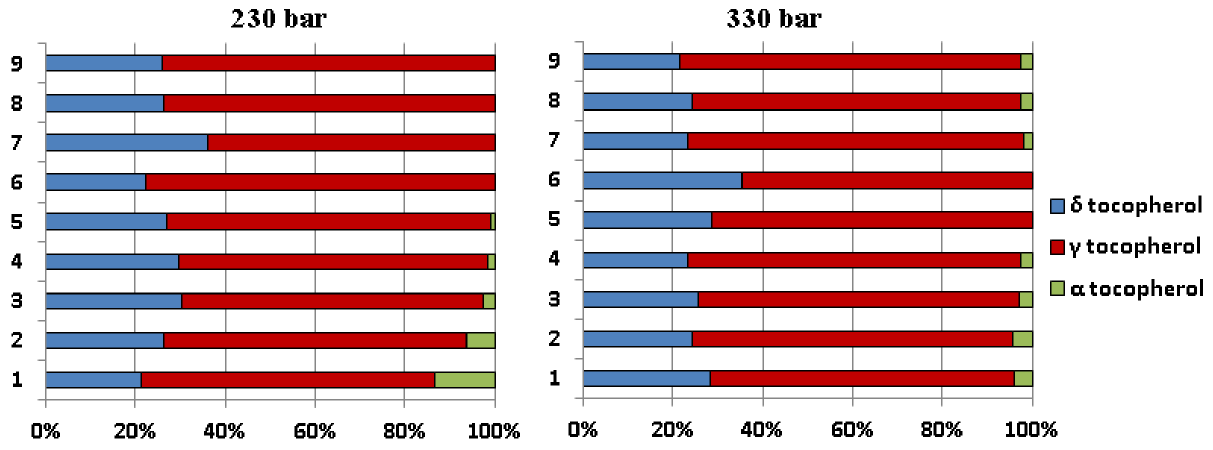

Figure S5. Percent distribution of tocopherols in oil fractions obtained using CO_2 at pressure of 230 and 330 bar.

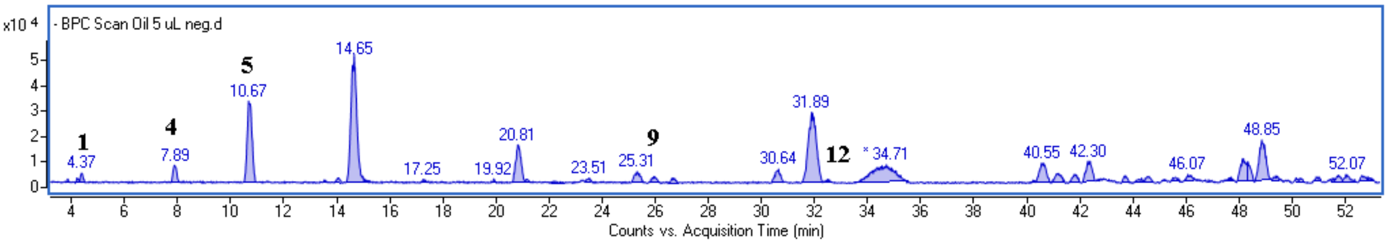

Figure S6. BPC chromatogram of oil sample obtained using CO_2 at pressure 230 bar (fraction F1). Numbering of components as in Table 3.

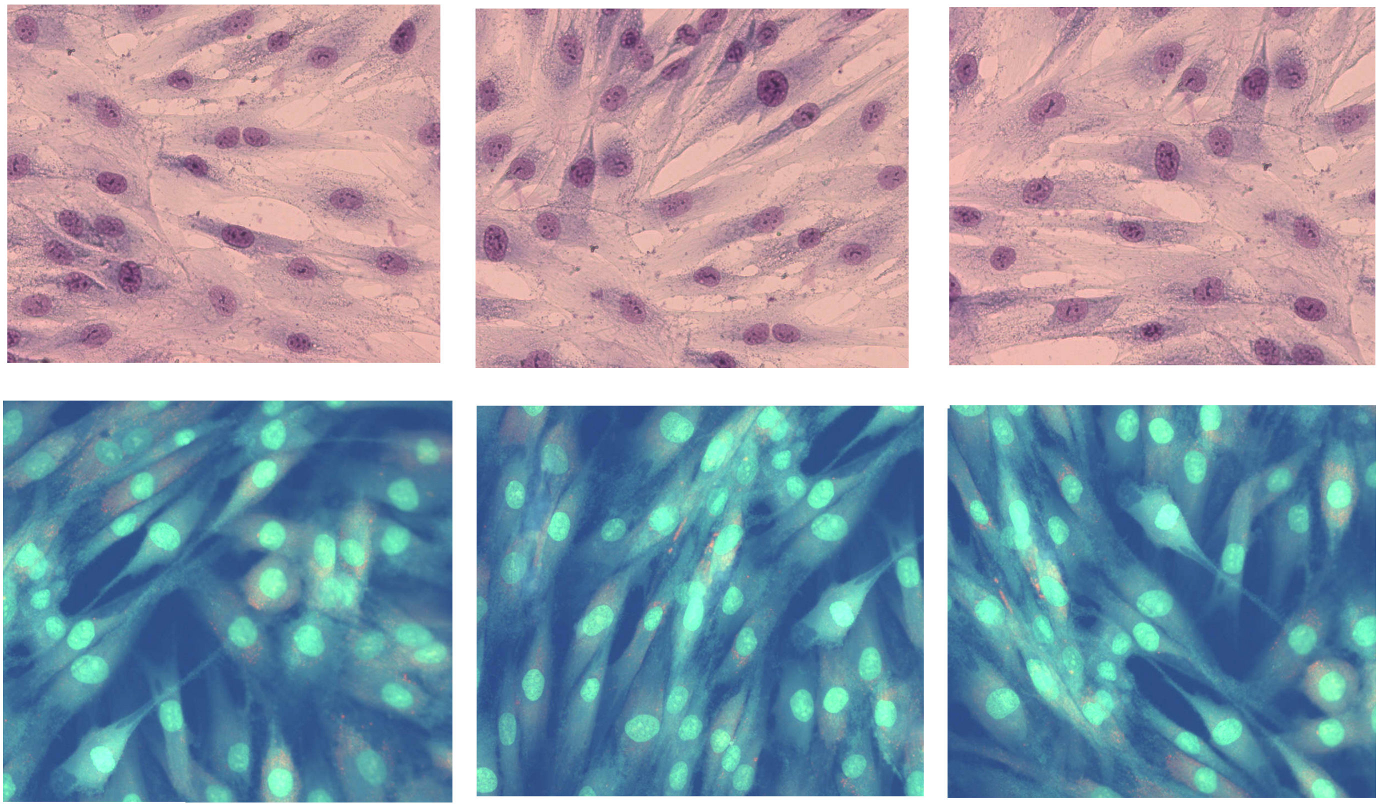

(a) (b) (c)

Figure S7. Image of human skin fibroblasts (HSF) cells: (a) control, (b) after 24-hour incubation with water/1,3-propanediol mixture (8/2 v/v) at a dilution of 1/80, (c) after 24-hour incubation with water/1,3-propanediol mixture (6/4 v/v) at a dilution of 1/80. May Grunwald-Giemsa and acridine orange staining (200x magnification).

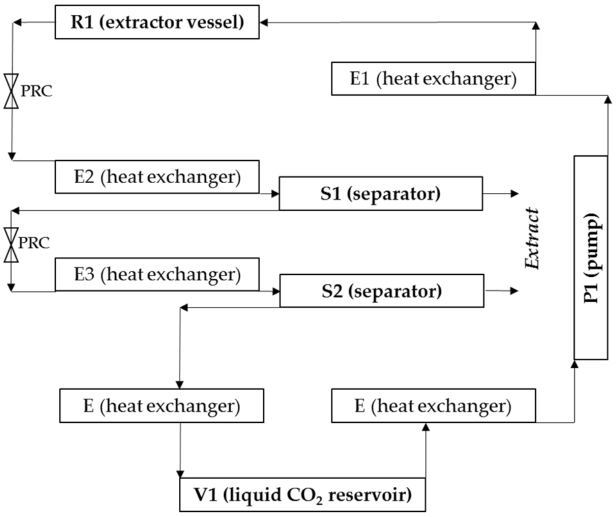

Figure S8. Block diagram of in-house built quarter-technical installation used in the study.

Table S1. UPLC-MS characteristic of compounds identified in methanol/water extract from blackcurrant seeds residues.

R_T [min]	$[M\text{-}H]^-$(fragments)	Δppm	Formula	Identified	Ref
4.49	169.01451 (125)	1.55	$C_7H_6O_5$	gallic acid	standard
6.00	299.07804 (137)	2.66	$C_{13}H_{16}O_8$	hydroxybenzoic acid hexoside	[1]
6.72	315.07287 (153)	2.26	$C_{13}H_{16}O_9$	dihydroxybenzoic acid hexoside	[1,2]
7.99	153.02004	4.60	$C_7H_6O_4$	protocatechuic acid	standard
10.82	137.02490	3.49	$C_7H_6O_3$	p-hydroxybenzoic acid	standard
15.75	197.03510	0.65	$C_9H_8O_4$	caffeic acid	standard
19.15	593.15321 (285)	3.39	$C_{27}H_{30}O_{15}$	Cyanidin rutoside	[3]
24.76	137.02477	2.55	$C_7H_6O_3$	Salicylic acid	standard
25.68	625.14334 (316)	3.70	$C_{27}H_{30}O_{17}$	Myricetin rhamnosylhexoside	[2]
26.24	479.08462 (316)	3.14	$C_{21}H_{20}O_{13}$	Myricetin 3-O-galactoside	standard
29.85	447.09265 (285)	-1.42	$C_{21}H_{20}O_{11}$	Kaempferol hexoside	[1]
31.57	609.14881	4.43	$C_{27}H_{30}O_{16}$	Quercetin rutoside	standard
32.51	463.09022 (300)	4.35	$C_{21}H_{20}O_{12}$	Quercetin 3-glucoside	standard
37.42	593.15261 (285)	2.38	$C_{27}H_{30}O_{15}$	Kaempferol-3-rutinoside	standard
38.29	447.09391 (285)	1.39	$C_{21}H_{20}O_{11}$	Kaempferol glucoside	standard
49.10	301.03681	4.75	$C_{15}H_{10}O_7$	Quercetin	standard
56.51	285.04069	0.8	$C_{15}H_{10}O_6$	Kaempferol	standard

1. Zhao, Y.; Lu, H.; Wang, Q.; Liu H.; Shen H.; Xu W.; Ge J.; He D. Rapid qualitative profiling and quantitative analysis of phenolics in Ribes meyeri leaves and their antioxidant and antidiabetic activities by HPLC-QTOF-MS/MS and UHPLC-MS/MS. *J. Sep. Sci.* **2021**, 44, 1404–1420.

2. D'Urso, G.; Montoro, P.; Piacente, S. Detection and comparison of phenolic compounds in different extracts of black currant leaves by liquid chromatography coupled with high-resolution ESI-LTQ-Orbitrap MS and high-sensitivity ESI-Qtrap MS. *J. Pharm. Bio.l Anal.*, **2020**, 179, 112926, https://doi.org/10.1016/j.jpba.2019.112926
3. Wu, X.; Gu, L.; Prior, R.L.; Mckay, S. Characterization of Anthocyanins and Proanthocyanidins in Some Cultivars of Ribes, Aronia, and Sambucus and Their Antioxidant Capacity, *J. Agric. Food Chem.* **2004**, 52, 7846-7856
